# Effect of a tailored assistive technology intervention on older adults and their family caregiver: a pragmatic study protocol

**DOI:** 10.1186/s12877-016-0269-3

**Published:** 2016-05-13

**Authors:** Louise Demers, W. Ben Mortenson, Marcus J. Fuhrer, Jeffrey W. Jutai, Michelle Plante, Jasmine Mah, Frank DeRuyter

**Affiliations:** Centre de recherche de l’Institut universitaire de gériatrie de Montréal, Centre intégré universitaire de santé et de services sociaux du Centre-Sud-de-l’Île-de-Montréal, Montréal, PQ Canada; École de réadaptation, Université de Montréal, Montréal, PQ Canada; Department of Occupational Science and Occupational Therapy, University of British Columbia, Vancouver, BC Canada; GF Strong Rehabilitation Research Program, Vancouver, BC Canada; International Collaboration on Repair Discovery, Vancouver, BC Canada; Eunice Kennedy Shriver National Institute of Child Health and Human Development, National Institutes of Health, Bethesda, MD USA; Interdisciplinary School of Health Sciences, University of Ottawa, Ottawa, ON Canada; Bruyère Research Institute, Ottawa, ON Canada; Department of Surgery/Speech Pathology and Audiology, Duke University, Durham, NC USA

**Keywords:** Randomized control trial, Older adults, Assistive technology, Informal caregiving

## Abstract

**Background:**

Many older adults with mobility limitations use assistive technology to help them perform daily activities. However, little attention has been paid to the impact on their family caregivers. This neglect produces an incomplete portrayal of the outcomes of assistive technology provision. This paper describes the protocol for a study that examines the impact of a tailored assistive technology intervention that is inclusive of assistance users and their family caregivers.

**Methods/design:**

This research will use a combination of quantitative and qualitative methods. The quantitative portion will be an experimental, single-blinded study in which participants are randomly assigned to either an experimental assistive technology intervention or a standard care group. We will enroll 240 participants (120 dyads) into the study from three Canadian sites. Participants will include older adults (>55) and family caregivers who provide ≥4 h per week of assistance with daily activities and social participation. The primary outcome measure for the older adults will be the Functional Autonomy Measurement System, and the primary outcome measure for the caregivers will be the Caregiver Assistive Technology Outcomes Measure. Qualitative data will be collected through detailed records of the therapists’ interventions, as well as through interviews with dyads and therapists following the interventions. Data collection will occur at baseline (T_0_) with follow-ups at 6 weeks (T_1_), 22 weeks (T_2_), and 58 weeks (T_3_) after baseline evaluation.

**Discussion:**

The findings from this study will help service providers and clinicians to move forward with assistive technology recommendations that are more attuned to the needs of both older adults with mobility limitations and their family caregivers. Additionally, the study’s findings will enhance our conceptual understanding of the spectrum of assistive technology outcomes and set the stage for econometric studies assessing cost-effectiveness.

**Trial registration:**

ClinicalTrials.gov Identifier: NCT01640470. Registered 11/21/2011.

## Background

With the aging population, there is an unprecedented growth of community-dwelling older adults experiencing physical disabilities and limitations of their mobility [1]. Assistive technology (AT) can play an important role in facilitating daily activities and social participation for these individuals [2]. Most commonly adopted AT include mobility-related items such canes, walkers, and wheelchairs, and environmental modifications such as grab bars, raised toilet seats, and bath seats [3, 4]. The use of these devices increases with age, ranging from 14–18 % in a healthy senior population [3] to 45–96 % in frail older adults [5, 6]. Notwithstanding this trend, large numbers of unmet AT needs have been reported in the ageing population [7, 8].

High quality evidence regarding the benefits of AT for older individuals is rare. One example is the randomized control trial (RCT) by Gitlin and colleagues [9]. This study found that the use of AT is associated with long-term positive outcomes with daily tasks, greater self-efficacy, less fear of falling, fewer home hazards, and greater use of adaptive strategies. Despite the potential benefits, adoption of AT by older adults is challenging. Common barriers include limited information about AT use and availability, its cost, and its incompatibility with users’ physical environment [10]. Another concern is the proportion of individuals who discontinue using the devices [11, 12]. To increase the acceptance and adherence to its optimal usage, greater attention to users’ and caregivers’ goals and preferences has been recommended [13, 14].

Insights on AT outcomes for older adults should also include consideration of the caregivers that provide them support. Indeed, a major goal for providing AT to older adults is that it decreases reliance on personal assistance or even replaces it [15–17]. In practice, both AT and human assistance are used by a majority of older people [16, 18–20]. This study is concerned with family caregivers, defined as individuals who provide unpaid assistance to individuals with disabilities [21, 22]. Family caregivers of older adults are frequently either spouses or adult children. In attempting to maintain or enhance the quality of life of those they help, those caregivers may experience a great deal of stress that can lead to their physical or emotional burnout [23]. The potential for burnout poses a challenge to the health care system, as family caregivers provide their unfunded assistance four times more frequently than formal caregivers [24–26]. This value excludes loss of economic productivity associated with time spent providing care, and emotional and physical burden [27].

Two conceptual models contribute to understanding the relationship between AT interventions and outcomes for family caregivers. The first model [28, 29] describes how assistance users’ personal strategies, which frequently include the use of AT, affect them and their family caregivers. Specifically, AT influences the manner and the extent of concerted human help required with activities. In some cases, a very successful outcome with AT may wholly eliminate the need for caregiver assistance, in terms of physical and psychological demands. The second model [30] demonstrates how AT can alter caregivers’ stressors so that their participation, health, and quality of life can be improved. The impact of AT varies with the type of device and the amount and manner in which it is used. The use of AT modulates the relationship among characteristics of primary stressors (e.g., areas of assistance, effort and safety), the features of secondary stressors (e.g., role overload and elective use of time), and broader caregiver outcomes (e.g., health and social participation).

Despite existing theory, little attention has been given to the impact of AT use on family caregivers in experimental studies. Mortenson and colleagues [17] conducted a systematic review suggesting that AT use reduces the degree of physical and emotional effort required when supporting an individual with a disability. However, the studies reviewed were primarily descriptive using cross-sectional data, undermining the strength of the inference. Additionally, the impact of AT use on caregivers was inferred from responses to very few queries, principally dealing with the number of hours of assistance provided. Marasinghe [16] reviewed existing findings to examine whether and how AT reduces caregiver burden. She found that AT contributes to reducing caregiver burden but that AT limitations can also add to their burden. In a previous study, our group [31] conduced a delayed intervention RCT with 44 dyads consisting of community-dwelling older adults and their family caregivers. It was the first experimental study to demonstrate that the provision of AT, embedded in an individually tailored approach, decreases caregiver burden and increases independence for older adults. The design did not, however, provide a parallel comparison group to assess the extent to which the intervention was superior to customary care and, thus, how it actually worked in real life. The findings from this RCT will respond to this gap and also aid in understanding the effectiveness of AT on improving older adults’ functional performance.

### Study goal and hypotheses

This RCT will examine whether a home-based, individually tailored approach to AT provision can improve older adults’ independence and decrease their family caregivers’ perceived burden compared to customary care. We expect that older adults randomized to the experimental condition will have higher activity performance following the novel intervention than those assigned to customary care, and that family caregivers will report significantly reduced burden in their caregiver activities.

*Hypothesis 1*: Compared to AT users in the customary care group, AT users in the experimental group will perform daily activities more independently after completion of the intervention. Secondarily, AT users in the experimental group will also perform instrumental activities of daily living more independently following the intervention.

*Hypothesis 2*: Following the intervention, family caregivers in the experimental group will report a significantly reduced frequency of physical and psychological demands associated with problematic caregiving activities, compared with caregivers in the customary care group. Secondarily, family caregivers in the experimental group will experience a decrease in their overall perceived burden following the intervention.

## Methods/design

The overall study will be based on a randomized controlled experimental design. However, a combination of qualitative and quantitative methods and measures will be used. The advantage of using mixed methods is that they provide multiple perspectives on an intervention’s outcomes so they can be understood more completely [32]. Multiple views also afford the opportunity for data triangulation, thus increasing the credibility of the findings. Throughout the course of administering the intervention, the therapists carrying out the experimental approach will record their impressions about both the successful and unsuccessful aspects of the intervention. A purposeful sample of dyads from both groups will be interviewed about their experiences after completing the intervention and at the conclusion of the study. Any changes to the protocol will be made through the trial registry. The anticipated end date of the study will be March 31, 2017.

### Quantitative study design

**Fig. 1:**
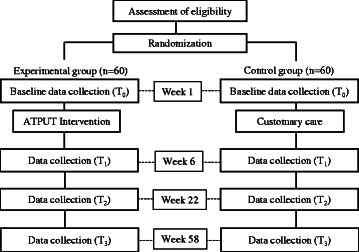
ATPUT, Assistive Technology Provision, Updating and Tune-Up intervention

This study protocol follows CONSORT guidelines. A single blind RCT will be conducted with 120 dyads, each consisting of an older AT user and his or her principal family caregiver. Both the experimental and customary care interventions will be provided to participants after the administration of the outcome measures at baseline. The measures will be re-administered to both groups 6 weeks, 22 weeks (the main trial endpoint), and 58 weeks after baseline evaluation. Figure 1 depicts the study design.

### Participants

Participants will be recruited from three sites in Canada: Montreal, Ottawa and Vancouver. To be eligible for the study, a dyad must consist of one assistance user and a one family caregiver. Assistance users must be (a) age 55 years or older, (b) living at home, (c) have a mobility limitation qualifying them for referral for homecare services, and (d) needing assistance with daily activities or social participation for a total of 4 h or more per week. Users may include individuals who currently are not using AT or are experiencing problems with their current devices. The population of interest is based off the amount of assistance received rather than on the diagnosis, because the use of AT is not diagnosis specific and there can be great heterogeneity among individuals with specific diagnoses. The family caregiver must be (a) age 18 years or older, and (b) delivering unpaid assistance with daily activities or social participation for a total of 4 h or more per week for at least 1 month. It is not required that assistance users and family caregivers reside together.

Dyads will be excluded if either member has a degree of cognitive impairment likely to prevent her or him from reliably completing the study questionnaires or if either has an advanced terminal illness. Cognitive status will be measured with the Montreal Cognitive Assessment (MoCA), a cognitive screening test designed to be sensitive to mild deficits. It has good test-retest reliability (correlation coefficient = .92) and internal consistency (Cronbach *α* = .83) [33].

### Recruitment

Participating dyads will be either existing clients or clients who have been recently referred to home care for occupational therapy services at the Montreal, Ottawa and Vancouver sites. Montreal participants will be recruited through the West Island Health and Social Services Center, Ottawa participants through the Community Care Access Centers, and Vancouver participants through the Vancouver Costal Health network. Candidates will be identified and recruited via referral lists and initially screened by home-care staff to ensure eligibility criteria are met. The sites’ study coordinators will then contact all interested individuals to confirm their eligibility, fully explain the project, answer questions, and obtain consent. Study participants will be permitted to withdraw from the intervention at any time they desire or if they become too ill. Due to ethical constraints the investigators cannot compel those who withdraw to provide further data. If the participant voluntarily discloses the reason for withdrawal, these will be documented.

### Randomization and blinding

Study coordinators at each site will randomly assign participants into the experimental group or control group immediately following their recruitment. The experimental group will consist of dyads that receive the home-based AT Provision, Updating and Tune-Up (ATPUT) intervention. Control group dyads will receive the customary care prevailing at the site where they were recruited. Centralized randomization software will be used to obtain the same number of participants in each group (www.randomization.com). Participating dyads will be blinded regarding their group assignment to the extent that they will be unfamiliar with the intervention received by the other group. Raters administering the outcome measures will be blinded until completion of the trial. Owing to the nature of the intervention, however, it is unfeasible to blind the study coordinators or the occupational therapists providing the intervention.

### Customary intervention

Current AT interventions in Canada take place in the community and are provided by registered occupational therapists. According to collaborators at the participating sites, customary interventions are not standardized. For example, family caregivers are not required to be included within the intervention process. Individuals are provided with equipment based on local funding policies and have limited practice time with devices. Follow-up visits are at the discretion of the therapists. Participants in the control group will receive services provided to clients by the health authorities within the study, with no additional intervention from the study.

### Experimental intervention

The experimental intervention will involve a detailed in-home assessment of the older adult’s current AT as well as the negotiation and implementation of a personal AT plan with the care recipient and his or her family caregiver. Conception of the home-based ATPUT intervention as a standardized approach to AT provision was influenced by the work of Roelands, Van Oost, Stevens, Depoorter, & Buysse [34] on the importance of shared decision-making by caregivers and AT users living in the community. It was developed in consultation with clinicians, AT users, and caregivers [29] and was found to be safe, feasible, and relevant to the targeted individuals when tested in a previous study [31]. It consists of 5 key steps described in Table 1.

**Table 1:** Steps in the Assistive Technology Provision Updating and Tune-Up Intervention

Step	Objectives	Intervention content
1. Identify and assess the problematic activities with involvement of both the family caregiver and recipient.	Agreement by the family caregiver and recipient on the choice of problematic activities to be targeted and features making them problematic.	Ascertain the difficulties related to the problematic activities and strategies currently used to deal with them.
		Observe how the activity is currently performed and determine the potential for assistance.
		Complete baseline assessment with tests targeting the skills required to perform the activity, if appropriate.
		Determine what can be improved.
Explore possible strategies to address the problematic activities.	Agreement among the family caregiver and recipient on an AT-related strategy to addressed the targeted problematic activities.	Discuss the family caregiver’s and older adult’s current strategies related to ATs.
		Describe the potential AT-related strategies (from simple to more complex);
		Discuss the advantages and disadvantages of strategies related to ATs in general and the targeted activity.
		Discuss their preferences in ways of doing things.
		Explore relevant characteristics of the physical and social environment of the dyad.
		Inform family caregiver and recipient of the skills required to use strategies related to ATs.
		Provide information and/or demonstrate candidate AT-related strategies (photos, scenarios).
		Give feedback in response to ideas/needs expressed by the dyad.
Choose most appropriate ATs solutions.	Agreement on the AT-related strategy to adopt.	Summarize information collected in the previous step.
		Inform family caregiver and recipient about relevant AT-related strategies available to them.
		Arrive at an agreement about the AT-related strategy to use
		Take steps to implement the strategy.
Conduct training.	Implementation of the AT-related strategy by the dyad and competence in utilizing the relevant AT.	Demonstrate the implementation of the AT-related strategy and use of the AT.
		Practice with family caregiver and care recipient.
		Provide feedback.
Evaluate effectiveness of the AT-related strategy.	Determine what additional intervention may be called for and/or motivate family caregiver’ and recipient’s to continue using the AT-related strategy.	Gauge the satisfaction of the family caregiver and recipient with performing the targeted activity and suggest remedies for problems that are noted.

In contrast to current standards of care, the ATPUT intervention will 1) follow a standardized multistep procedure that will be documented during each encounter using a study-specific tool, 2) include additional in-person follow-up visits that are not necessarily part of customary care, 3) involve the AT user and family caregiver collaboratively, and 4) provide AT and AT funding in a timely manner, entailing financial assistance to repair or acquire new AT, and training. In this regard, the frequency and intensity of the experimental intervention is expected to be greater than customary care. Participants will be allowed to pursue complimentary care during the course of the ATPUT intervention. These will be documented in the life changes form.

The intervention will be delivered by registered occupational therapists trained by the research team to use the same standardized approach and materials, based on a treatment manual [31]. To avoid possible contamination, therapists who deliver the intervention to the experimental group will not treat members of the control group.

### Treatment fidelity

We will follow the NIH Behavior Change Consortium’s recommendations [35] for enhancing treatment fidelity. We address considerations of study design (intervention is distinct, based on a conceptual framework), provider training (occupational therapists will be trained on the ATPUT through in-person or video-conferencing, and will be using an intervention manual), treatment delivery (reminder calls before each visit, home environment where the skill is actually performed, treatment planning by the occupational therapists), treatment receipt (checklist of all intervention operations with dates performed), and enactment of treatment (checks if the AT was obtained and if necessary, installed; evaluation of the AT solution during the final visit; adherence form to document the degree of adherence to recommendations). Coordinators will monitor checklists and review interventionist data collection sheets for possible errors and coherence. They will provide individual feedback regularly to occupational therapists and collect their notes, reflections and comments.

### Quantitative measurements

Table 2 highlights the measures to be administered at each time point. Raters will administer all quantitative measures. They will be trained by the study coordinator to ensure standardized administration of the measures. The primary outcome measure for care recipients will be a composite score from two sub-scales of the Functional Autonomy Measurement System (SMAF) [36]. These sub-scales will include self-care and mobility, and exclude the communication and mental functioning sub-scales that are unlikely to be affected by the ATPUT intervention. The SMAF will be administered to care recipients and caregivers together, but all other outcome measures will be administered to them separately. The secondary outcomes will be the self-administered Functional Independence Measure (FIM), a measure of self-assessed independence in daily activities [37, 38], and the Reintegration to Normal Living Index (RNLI), a measure of social participation that address daily activities, roles and relationships [39, 40]. Finally, the SMAF sub-scale relating to instrumental activities of daily living will also be used as a secondary outcome.

**Table 2:** Quantitative measures and assessments collected at baseline, week 6, week 22 and week 58

Measures	Tools/Metrics	Baseline	6 weeks	22 weeks	58 weeks
Primary outcomes for AT users	SMAF sub-scales, self-care & mobility	X	X	X	X
Secondary outcomes for AT users	Self-report FIM RNLI EQ-5D SMAF sub-scale, IADL	X	X	X	X
Primary outcomes for family caregivers	CATOM (items 1–14)	X	X	X	X
Secondary outcomes for family caregivers	CATOM (items 15–18) CBI Hours of care	X	X	X	X
Socio-demographic and clinical data for AT users	MoCA^a^ Study-specific questionnaire	X			
Socio-demographic and clinical data for family caregivers	Study-specific questionnaire	X	X	X	X
Treatment fidelity and Intervention information	Adherence Form Life Changes Form Chart review^b^ Therapist survey^b^		X	X	X

*Abbreviations*: *CATOM* Caregiver Assistive Technology Outcome Measurement, *CBI* Caregiver Burden Inventory, *EQ-5D* European Quality of Life, *FIM* Functional Independence Measure, *IADL* Instrumental activity of daily living, *MoCA* Montreal Cognitive Assessment, *RNLI* Reintegration to Normal Living Index, *SMAF* Functional Autonomy Measurement System

^a^MoCA is only re-administered at T1, T2, and T3 if major changes are observed by the raters

^b^Chart reviews and therapist surveys are done at 6 weeks or later

The primary outcome measure for caregivers will be the Caregiver AT Outcome Measure (CATOM) [31, 41]. Construction of the CATOM was based on a conceptual framework of AT outcomes for AT users and their caregivers [30]. The first part (items 1–14) classifies specific activities for which the caregiver provides support, and then measures the frequency of caregiving and the perceived burden associated with the activities (e.g., feeling physically tired after helping with the specified activities). The original CATOM was designed to measure outcomes produced by AT interventions for a single problematic activity, whereas the current study will use a revised version that permits tracking the effects of AT interventions for multiple problematic activities. In the original study, the internal consistency of the activity-specific section of the CATOM was *α* = .73 [31]. The second part of the CATOM measures overall burden and will be used as a secondary outcome measure. The overall burden section is comprised of four items (15–18) measuring the caregiver’s degree of burden associated with all of the assistance provided (e.g., feeling that caregiving duties limits recreational and leisure activities). The internal consistency of this section was *α* = .78 [31]. Other secondary outcomes for caregivers will include hours of weekly care provided, the Caregiver Burden Inventory (CBI) [42], and the European Quality of Life (EQ-5D) visual analog system and descriptive system [43]. The EQ-5D measures the caregivers’ health status; the descriptive system is comprised of 5 items scored from 1 to 3, with higher total scores indicating worse health. The CBI provides a comparative measure of general caregiver burden whereas the CATOM focuses on caregiving as it relates to AT.

Socio-demographic and clinical data will be collected about older adults’ age, sex, level of education, ethnic origin, language, marital status, type of dwelling, diagnoses, duration of functional problems, and amount of formal caregiving received, if applicable. For family caregivers, collected data will include age, sex, level of education, language, the relationship with the older adult and cohabitation (yes/no), employment (yes/no), and duration of assistance.

An adherence form and life changes form will be administered respectively to capture the degree of adherence to occupational therapist recommendations and changing events in the lives of dyad members that could affect outcome measures. Older adults’ and family caregivers’ versions will be administered separately. The life changes form will also capture any adverse events that occur. A chart review of the occupational therapist notes for each dyad will summarize the reasons for referral, the number of visits, the AT provided, and the goals of each session. This review will be conducted after the interventions (customary or experimental) are completed, i.e., six weeks or more following baseline assessment. A therapist survey will also be completed by participating occupational therapists to collect data about their level of education, years of practice, and years of experience working in this area.

### Quantitative sample size

Sample size was calculated for the primary endpoint (22 weeks) for older adults and family caregivers separately and the largest sample size was selected. The sample size for the primary caregiver outcome measure (CATOM) was chosen to detect a 10 % decrease in perceived burden. This is consistent with the caregiver burden change noted in a prior trial using CATOM scores at baseline and after 16 weeks [31]. It indicates a one-level decrease on half of the items for the family caregivers (nearly always → frequently → sometimes → rarely → not at all). The primary older adults’ outcome measure is the SMAF. The sample size was chosen in order to detect an improvement of 9 % following the intervention. This value is intermediate between the minimal detectable difference and the change measured by administering the SMAF in a study examining the impact of a rehabilitation program on individuals with post-cerebral vascular accident [44, 45].

To determine an effect size, Cohen’s *D* was calculated using standard deviations from our prior study [31] and then transformed into Cohen’s *F* [46, 47]. As the effect size was smaller for the SMAF, we used this value to simulate various potential scenarios using G*Power 3.1.0.87. For these simulations we varied several variables including 1) effect size (0.13 to 0.15), 2) correlations among repeated measures (which previous data suggest will be moderate for pre-post intervention and strong between post intervention and long term follow up, e.g. 0.5 to 0.6), and 3) violations of sphericity (due to differences in variances over time, e.g., 0.6 to 0.9). The average of these sample sizes is 102. Based on approximately a 20 % dropout rate for the main trial endpoint (suggested by the prior study in which dropout occurred exclusively due to health related issues, which are common in this population), a sample of 120 dyads will be recruited (40 from each site).

### Quantitative data analysis of primary and secondary outcome measures

Data will be entered by raters into SPSS statistics data editor (IBM SPSS Statistics) immediately after each evaluation session to decrease the likelihood of missing data. The use of multiple imputation will be explored to deal with any missing data, especially if a participant is unable to be assessed at one of the data collection time-points [48].

To ensure baseline similarity between groups, the experimental and control groups will be compared on socio-demographic and clinical variables with the use of *t*-tests for continuous data and Chi-square for nominal data. Descriptive statistics and histograms will be used to display differences between the groups. A repeated measures analysis of variance will be conducted to analyze dependent variable data, using a Greenhouse-Geisser correction to address issues with sphericity.

Scores for the primary outcome measures (SMAF and CATOM, items 1–14) will be compared between treatment groups over time using an intention-to-treat analysis. Intention-to-treat analysis dictates that all participants are included in the group to which they were allocated for purposes of analysis, whether or not they completed the intervention for that group. The same analysis will be applied to secondary outcome measures, which include the RNLI, CATOM (items 15–18), CBI, self-report FIM and hours of care provided by family caregivers. These secondary outcome analyses will be considered exploratory in view of the increased likelihood of Type I errors resulting from multiple statistical comparisons. Sensitivity analyses will be performed to compare the effect of the interventions on 1) caregiving spouses versus caregiving adult children, and 2) on participants receiving services at the different sites.

To test for changes in family caregiver burden due to the intervention, changes in older adults’ SMAF scores will be correlated with changes in their caregivers’ CATOM scores, using either a Pearson or Spearman correlation depending on the distribution of the data.

### Quantitative data analysis for treatment fidelity

The percentage of intervention protocol items completed for each dyad in the experimental group will be calculated to determine treatment fidelity. These data will be supplemented with quantitative data retrieved from the adherence form, life change forms and chart reviews.

### Quantitative data analysis for intervention information

For both groups, descriptive statistics will be used to characterize therapists’ number, length, and total time of visits; the number, types, and costs of devices; and occupational therapists’ qualifications, years of practice, and years of experience working in this area. Exploratory post-hoc analysis will examine how these intervention elements may have contributed to the various results such as therapist differences, intensity of intervention, delay in receipt of equipment, and types of devices provided.

### Participants for the qualitative study

To obtain variation within our interview sample [49], participants will differ in terms of 1) type of family caregivers, e.g., spouses or adult children; 2) older adults’ baseline functional autonomy levels (based on SMAF scores); 3) type of AT provided; 4) research sites, and 5) experimental versus control group membership. Based on our previous research with this population and suggested sample sizes for such research [50], we expect to conduct interviews with 20 to 25 dyads (40–50 individual interviews). As a sub-sample of the larger study, interview participants will meet all of the inclusion criteria for the quantitative study described above. As part of the consent process, we will indicate that participants in the subsample may be asked to take part in two additional qualitative interviews.

### Qualitative measures and data collection

To better understand how the intervention was administered by therapists and experienced by older adults and their family caregivers, three systematic qualitative methods of data collection will be used. First, the registered occupational therapists providing the experimental intervention will keep a record of the interventions that they provide. The records will be reflective in nature and describe a) the content of the treatment session, b) elements of the intervention that seem to be working well, c) aspects that could be improved and d) general impressions. Second, therapists from each site, regardless of whether they administered care to the experimental or control group, will be invited to be interviewed individually to determine the process they used when providing AT. Third, qualitative interviews will be conducted with a purposeful selection of AT users and family caregivers, to refine emerging interpretation of the experience throughout the study. A member of the research team who was not involved in data collection or treatment administration will conduct these interviews. They will be scheduled following quantitative data collection at week 6 and at the end of the study.

### Qualitative data analysis

Qualitative interviews will be digitally recorded and transcribed verbatim. The transcripts will be reviewed while listening to the audio file to ensure accuracy. Each interview transcript and audio files will be labeled and stored in qualitative data analysis software (NVivo10) according to a) the participant’s pseudonym, b) the type of participant (occupational therapist, family caregiver or AT user), c) the group type (control or experimental), and d) its location (Montreal, Ottawa or Vancouver).

By repeatedly reviewing the data, we will develop ideas and interpretations about recurring, converging and contradictory patterns and will identify key concepts and themes along with illustrative examples [51]. Broad categories will be developed to organize and inductively code the raw data. Themes within and across participants are likely to emerge by means of this iterative process [50]. Examples from the themes will be retrieved and compared between and within field notes, participant observations, and interview transcripts. As the review progresses, data will be coded and re-coded to reflect emergent themes. “Negative cases” that do not fit with the emergent themes will be explored to develop tentative explanations for them. Ultimately, codes will be grouped into relevant themes, and they will be organized in a manner that is intended to promote understanding of how the experimental and control interventions were experienced.

Results from the treatment reflections and qualitative interviews will be compared with the quantitative results for older adults and their family caregivers to look for divergent and complementary findings. The results for each member of the dyad will be scrutinized separately, as will data for the dyads comprising the experimental group, especially when exploring interaction effects between older adults and family caregivers. Qualitative and quantitative data will also be compared for individual participants.

Given the previously demonstrated efficacy of AT in earlier RCT interventions [9, 31, 52, 53], no data monitoring committee (DMC) will be used. The final qualitative and quantitative trial dataset produced by the intervention will be managed by and accessible to study investigators, only. The dissemination of all findings and analysis will be through academic articles authored by the investigators that are published in refereed open access journals. Participants who express interest will receive a lay summary of the findings of the study.

## Discussion

As a society, we need all the empirical, methodological, and theoretical knowledge available to respond adequately to the problems faced by older adults, their family caregivers, and service providers with respect to the efficient use of assistive technology. This study will be the first parallel-group RCT to examine an AT intervention with emphasis on both the older adult with disabilities and his/her family caregiver. Providing evidence of the effectiveness of such an approach will encourage service providers and clinicians to move forward with AT recommendations that are more attuned to the needs of both members of the dyad. It is important to recognize the societal importance of responding to the needs of family caregivers who in many instances may be aging themselves. Resource limitations and social policies that promote aging in place also bolster the call for an adequate response to family caregivers’ needs. Positive findings will highlight the value of an approach to AT provision that is more inclusive of family caregivers and will set the stage for econometric studies assessing its cost-effectiveness.

The study is designed to evaluate the effectiveness of an AT intervention in real-life conditions. As such, the results are prone to be influenced by a host of factors including older adults’ and family caregivers’ clinical and socio-demographic characteristics, their home-related problematic activities, their home environment, the device types they are supplied, and the dynamics of the relationship between them. This places a premium on having sufficient power to control for the concomitant effects of those factors. However, this study has the advantage of being built on the results of a previous one, which did detect a clinically significant effect of the ATPUT intervention [31]. Moving from that study to this planned, parallel-group RCT is a logical step toward the next best evidence. The results from the planned study should be transferable and usable in real life.

Our design combines qualitative and quantitative methods and measures. Given the limited research on the relationship between caregiver burden and AT, the qualitative data will extend our knowledge in this area beyond the results of the quantitative portion of the research and will allow exploration of possible feelings of ambivalence toward assistive devices that have been reported in previous studies [54, 55]. The proposed research approach may serve to highlight ways of improving the way AT and AT education is provided in the community, and may suggest a model for assessing the effectiveness of other AT interventions. It may also advance the methodology of studying AT outcomes for AT users and caregivers.

## Ethics approval and consent to participate

The research protocol to be used in this study has been approved by the offices of research ethics boards of the participating sites: Institut Universitaire de Gériatrie de Montréal (reference 11-12-018), West Island Health and Social Services Center (no reference number), University of Ottawa (reference A09-11-02), Bruyère Continuing Care (reference M16-11-021), University of British Columbia – Providence Health Care Research Institute (reference H12-03195), and Simon Fraser University (reference 2011 s0346). All participants consented to be involved in the study.

## Consent for publication

Not applicable.

## Availability of data and materials

The dataset(s) supporting the conclusions of this article are available to the public and can be obtained by contacting the first author.

## Abbreviations

AT: assistive technology
ATPUT: Assistive Technology Provision, Updating and Tune-Up
CATOM: Caregiver Assistive Technology Outcome Measure
CBI: Caregiver Burden Inventory
EQ-5D: European Quality of Life
FIM: Functional Independence Measure
MoCA: Montreal Cognitive Assessment
RCT: randomized control trial
RNLI: Reintegration to Normal Living Index
SMAF: Functional Autonomy Measurement System

## References

[1] Kaye HS (2013). Disability rates for working-age adults and for the elderly have stabilized, but trends for each mean different results for costs. Health Aff (Millwood).

[2] Cook AM, Polgar JM (2014). Assistive technologies: principles and practice.

[3] Cornman JC, Freedman VA, Agree EM (2005). Measurement of assistive device use: Implications for estimates of device use and disability in late life. Gerontologist.

[4] Gitlin LN (2002). Assistive technology in the home and community for older people: psychological and social considerations. In Scherer MJ, editor. Assistive technology: matching device and consumer for successful rehabilitation.

[5] Kaye HS, Yeager P, Reed M (2008). Disparities in usage of assistive technology among people with disabilities. Assist Technol.

[6] McCreadie C, Tinker A. The acceptability of assistive technology to older people. Ageing Soc. 2005. doi.10.1017/S0144686X0400248X

[7] Gramstad A, Storli SL, Hamran T (2013). “Do I need it? Do I really need it?” elderly peoples experiences of unmet assistive technology device needs. Disabil Rehabil Assist Technol.

[8] Statistics Canada (2008). Participation and activity limitation survey 2006: a profile of assistive technology for people with disabilities.

[9] Gitlin LN, Winter L, Dennis MP, Corcoran M, Schinfeld S, Hauck WW (2006). A randomized trial of a multicomponent home intervention to reduce functional difficulties in older adults. J Am Geriatr Soc.

[10] Ahn M, Beamish JO, Goss RC. Understanding older adults’ attitudes and adoption of residential technologies. Fam. Consum. Sci. Res. J. 2008. doi:10.1177/1077727X07311504

[11] Demers L, Fuhrer MJ, Jutai JW, Scherer MJ, Pervieux I, DeRuyter F (2008). Tracking mobility-related assistive technology in an outcomes study. Assist Technol.

[12] Phillips B, Zhao H (1993). Predictors of assistive technology abandonment. Assist Technol.

[13] Dijcks BP, De Witte LP, Gelderblom GJ, Wessels RD, Soede M (2006). Non-use of assistive technology in the Netherlands: a non-issue?. Disabil Rehabil Assist Technol.

[14] Fuhrer M, Jutai J, Demers L, Scherer M, Bloch E, DeRuyter F. Effects of type of locomotive device and disabling condition on device use and disuse among elderly individuals following hospitalization. Proceedings of the International Conference of Aging, Disability and Independence. St-Petersburg, FL; 2006

[15] Hammel J, Burdick D, Kwon S (2004). Assistive technology as tools for everyday living and community participation while aging. Gerontechnology: research and practice in technology and aging.

[16] Marasinghe KM. Assistive technologies in reducing caregiver burden among informal caregivers of older adults: a systematic review. Disabil Rehabil Assist Technol. 2015. doi:10.3109/17483107.2015.1087061 10.3109/17483107.2015.1087061 26371519

[17] Mortenson WB, Demers L, Fuhrer MJ, Jutai JW, Lenker J, DeRuyter F (2012). How assistive technology use by individuals with disabilities impacts their caregivers: a systematic review of the research evidence. Am J Phys Med Rehabil.

[18] Agree EM, Freedman VA, Cornman JC, Wolf DA, Marcotte JE (2005). Reconsidering substitution in long-term care: when does assistive technology take the place of personal care?. J Gerontol B Psychol Sci Soc Sci.

[19] Allen S, Foster A, Berg K (2001). Receiving help at home: the interplay of human and technological assistance. J Gerontol B Psychol Sci Soc Sci.

[20] Taylor DH, Hoenig H (2004). The effect of equipment usage and residual task difficulty on use of personal assistance, days in bed, and nursing home placement. J Am Geriatr Soc.

[21] Arno PS, Levine C, Memmott, MM. The economic value of informal caregiving. Health Aff (Millwood). 1999. doi:10.1377/hlthaff.18.2.182 10.1377/hlthaff.18.2.182 10091447

[22] Zukewich N (2003). Unpaid informal caregiving. Canadian Social Trends.

[23] Schulz R, Martire LM, Klinger JN (2005). Evidence-based caregiver interventions in geriatric psychiatry. Psychiatr Clin North Am.

[24] Agree EM, Freedman VA, Sengupta M (2004). Factors influencing the use of mobility technology in community-based long-term care. J Aging Health.

[25] Fast J, Niehaus L, Eales J, Keating N (2002). A profile of Canadian palliative care providers. Research on aging, policies and practice.

[26] Health Council of Canada. Seniors in need, caregivers in distress: What are the home care priorities for seniors in Canada? Toronto: Health Council of Canada; 2012. http://www.alzheimer.ca/durham/~/media/Files/on/Media%20Releases/2012/April%202012/HCC_HomeCare_2d.pdf

[27] Fast J, Eales J, Keating N (2001). Economic impact of health, income security and labour policies on informal caregivers of frail seniors.

[28] Demers L, Fuhrer MJ, Jutai JW, Lenker JA, DeRuyter F. A framework for evaluating assistive technology outcomes on the user-caregiver dyad. Festival of International Conferences on Caregiving, Disability, Ageing, and Technology (FICCDAT) – Festival Proceedings 2007 [CD-ROM], T0076.

[29] Demers L, Mortenson WB, Scherer M, Federici S (2012). Measuring the impact of assistive technology on family caregivers. Assistive technology assessment handbook.

[30] Demers L, Fuhrer MJ, Jutai J, Lenker J, Depa M, DeRuyter F (2009). A conceptual framework of outcomes for caregivers of assistive technology users. Am J Phys Med Rehabil.

[31] Mortenson WB, Demers L, Fuhrer MJ, Jutai JW, Lenker J, DeRuyter F (2013). Effects of an assistive technology intervention on older adults with disabilities and their informal caregivers. An exploratory randomized controlled trial. Am J Phys Med Rehabil.

[32] Wisdom J, Creswell JW (2013). Mixed methods: integrating quantitative and qualitative data collection and analysis while studying patient-centered medical home models.

[33] Nasreddine ZS, Phillips NA, Bédirian V, Charbonneau S, Whitehead V, Collin I (2005). The Montreal cognitive assessment, MoCA: a brief screening tool for mild cognitive impairment. J Am Geriatr Soc.

[34] Roelands M, Van Oost P, Stevens V, Depoorter AM, Buysse A (2004). Clinical practice guidelines to improve shared decision-making about assistive device use in home care: a pilot intervention study. Patient Educ Couns.

[35] Bellg AJ, Borrelli B, Resnick B, Hecht J, Minicucci DS, Ory M (2004). Enhancing treatment fidelity in health behavior change studies: best practices and recommendations from the NIH behavior change consortium. Health Psychol.

[36] Desrosiers J, Bravo G, Hébert R, Dubuc N (1995). Reliability of the revised functional autonomy measurement system (SMAF) for epidemiological research. Age Ageing.

[37] Jensen MP, Abresch RT, Carter GT (2005). The reliability and validity of a self-report version of the FIM instrument in persons with neuromuscular disease and chronic pain. Arch Phys Med Rehabil.

[38] Masedo AI, Hanley M, Jensen MP, Ehde D, Cardenas DD. Reliability and validity of a self-report FIM™ (FIM-SR) in persons with amputation or spinal cord injury and chronic pain. Am J Phys Med Rehabil. 2005. doi:10.1097/01.PHM.0000154898.25609.4A 10.1097/01.phm.0000154898.25609.4a 15725790

[39] Wood-Dauphinee SL, Williams JI (1987). Reintegration to normal living as a proxy to quality of life. J Chronic Dis.

[40] Wood-Dauphinee SL, Opzoomer A, Williams JI, Marchand B, Spitzer WO (1988). Assessment of global function: the reintegration to normal living index. Arch Phys Med Rehabil.

[41] Mortenson WB, Demers L, Fuhrer MJ, Jutai JW, Lenker J, DeRuyter F (2015). Development and preliminary evaluation of the caregiver assistive technology outcome measure. J Rehabil Med.

[42] Novak M, Guest C (1989). Application of a multidimensional caregiver burden inventory. The Gerontologist.

[43] The EuroQol Group (1990). EuroQol – a new facility for the measurement of health-related quality of life. Health Policy.

[44] Demers L, Desrosiers J, Nikolova R, Robichaud L, Bravo G (2010). Responsiveness of mobility, daily living, and instrumental activities of daily living outcome measures for geriatric rehabilitation. Arch Phys Med Rehabil.

[45] Desrosiers J, Rochette A, Noreau L, Bravo G, Hébert R, Boutin C (2003). Comparison of two functional independence scales with a participation measure in post-stroke rehabilitation. Arch Gerontol Geriatr.

[46] Cohen J (1998). Statistical power analysis for the behavioural sciences.

[47] Faul F, Erdfelder E, Lang AG, Buchner A (2007). G^* power 3: a flexible statistical power analysis program for the social, behavioral, and biomedical sciences. Behav Res Methods.

[48] Kenward MG, Carpenter J (2007). Multiple imputation: current perspectives. Stat Methods Med Res.

[49] Sandelowski M. Sample size in qualitative research. Res. Nurs. Health. 1995. doi:10.1002/nur.4770180211 10.1002/nur.4770180211 7899572

[50] Morse JM, Field PA (1995). Nursing research: the application of qualitative approaches.

[51] Hammersley M, Atkinson P. Ethnography: principles in practice. 3rd ed. New York: Routledge; 2007.

[52] Mann WC, Ottenbacher KJ, Fraas L, Tomita M, Granger CV (1999). Effectiveness of assistive technology and environmental interventions in maintaining independence and reducing home care costs for the frail elderly. A randomized controlled trial. Arch Fam Med.

[53] Wilson DJ, Mitchell JM, Kemp BJ, Adkins RH, Mann WC (2009). Effects of assistive technology on functional decline in people aging with a disability. Assist Technol.

[54] Pettersson I, Berndtsson I, Appelros P, Ahlström G (2005). Lifeworld perspectives on assistive devices: lived experiences of spouses of persons with stroke. Scand J Occup Ther.

[55] Rudman DL, Hebert D, Reid D (2006). Living in a restricted occupational world: the occupational experiences of stroke survivors who are wheelchair users and their caregivers. Can J Occup Ther.

